# Women’s attitude towards umbilical cord blood banking in Poland

**DOI:** 10.1007/s10561-021-09914-y

**Published:** 2021-03-22

**Authors:** Agata Pisula, Agnieszka Sienicka, Karolina Stachyra, Joanna Kacperczyk-Bartnik, Paweł Bartnik, Agnieszka Dobrowolska-Redo, Ewa Romejko-Wolniewicz

**Affiliations:** 1 Students’ Scientific Group Affiliated to 2nd Department of Obstetrics and Gynecology, Medical University of Warsaw, Warsaw, Poland; 2 2nd Department of Obstetrics and Gynecology, Medical University of Warsaw, Karowa 2 St., 00-315 Warsaw, Poland

**Keywords:** Blood banks, Cord blood stem cell transplantation, Fetal blood

## Abstract

Umbilical cord blood (UCB) is considered as a valuable potential source of hematopoietic stem and progenitor cells. A process of collecting and storing UCB in the immediate period after the birth is called UCB banking. The study was conducted in order to determine women’s knowledge, awareness, preferences and attitude towards UCB banking in Poland, considering the sociodemographic and obstetric factors. A cross-sectional, self-administered, online questionnaire-based study including mostly multiple choice questions concerning attitude and awareness regarding UCB banking was conducted entirely online among Facebook female users in Poland. A total of 1077 participants correctly completed the survey. Most participants (n = 911, 84.6%) were aware of the possibility of UCB banking. Social media were considered as the main source of information (47.5%). However, the participants mostly indicated the doctor as their preferred source of reliable information (86.8%). The majority of women (61.8%) assessed their level of knowledge of UCB banking as still insufficient. Among the participants who supported UCB banking (70%), the following reasons were considered as the most vital: potential possibility of helping their child (93.9%) and helping other relatives (64.4%). More than half of the respondents (66.9%), who have not stored and are not willing to store their children’s UCB, indicated the high cost of UCB banking as the main reason of this decision. The knowledge and awareness of UCB storage and banking possibilities amongst women in Poland could be improved. The professional medical personnel should be a source of reliable information.

## Introduction

Umbilical cord blood (UCB) is considered as a valuable potential source of hematopoietic stem and progenitor cells (Lee et al. 2010). Umbilical cord blood banking is a relatively new technique gaining popularity in the past 30 years. The first successful umbilical cord blood transplantation dates back to 1988. It was performed in France in a child with Fanconi Anaemia (FA) (Ballen et al. 2013). In Poland, the first cord blood stem cell transplant took place in 1994 to treat a boy suffering from acute myelogenous leukaemia (Borys-Wójcik et al. 2019). In 2007, there was the first Polish transplant of umbilical cord blood which was previously collected, processed and stored in a private bank (Borys-Wójcik et al. 2019; PBKM 2007). Since then, umbilical cord blood stem cells have been transplanted over 40.000 times worldwide including a few transplants in Poland (Ballen 2017; FamiCord Group 2020).

Umbilical cord blood banking is a process of collecting and storing umbilical cord blood in the immediate period after the birth. Two umbilical cord blood banking options are available: private (commonly known as family) and public. The first one, where parents are charged a fee for the collection, processing and storage of their infant’s umbilical cord blood, UCB is available exclusively for the autologous or family use. In public banks umbilical cord blood can be donated without financial cost. However, the UCB unit is not reserved for the use of donating family (Peberdy et al. 2018). In Poland, there are 8 public and 5 private UCB banks. The cost of cord blood collection in Polish private banks ranges from 1500 to 2500 PLN and each subsequent year of blood storage costs about 500 PLN (EUROCORD 2020a, b). The most recent data concerning the amount of umbilical cord blood stored in Polish banks comes from 2016. According to this, at the end of 2015 there were about 113.500 portions of UCB stored in private banks and about 4.300 portions in public ones (Uhrynowska-Tyszkiewicz 2016).

## Objectives

The study was conducted in order to determine women’s knowledge, awareness, preferences and attitude towards umbilical cord blood banking in Poland, considering the sociodemographic and obstetric factors.

## Materials and methods

The questionnaire-based study was conducted to elicit and analyse the attitudes and knowledge about the UCB collecting procedure and banking among women in Poland. The self-administered and voluntary online survey was distributed on 46 Polish Facebook groups. The questionnaire was designed using the survey administration application—Google Forms. The anonymity and confidentiality were ensured. The participants were random women from Poland who were members of social media groups. The data were collected for approximately one month, between 2nd December and 28th December 2019. The developed questionnaire mostly included the multiple choice questions. It was divided into 4 sections. Within the first part the respondents self-reported the sociodemographic factors including age, education, marital status or household income. In the second one they were asked about their obstetric history regarding the pregnancy and delivery topics. The third and fourth parts gathered information about the source of their knowledge about the usage of stem cells, their attitude and awareness regarding UCB. They were to ascertain their expectations about UCB banking as well as their view on private and public institutions. If somebody had never heard of UCB banking, the questionnaire would have been finished after the third section.

In order to achieve 99% confidence level and 4% margin of error, the sample size of the population of women was calculated to be 1041 participants.

Obtained data were analysed using descriptive statistics and chi-square test for categorical variables. The findings were considered statistically significant if p value was less than 0.05.

The datasets generated during and/or analysed during the current study are available from the corresponding author on reasonable request.

## Results

A total of 1077 women participated in the study. All of the answers were correctly completed and used for the further analysis. As mentioned before, women (n = 166, 15.4%), who had never heard of the UCB banking before, finished their questionnaire after the third section.

Table 1 includes the sociodemographic factors of all 1077 participants. More than half of them have a higher education and live in a big city.

**Table 1:** Sociodemographic characteristics of participants

Category	Variables	Number	Percentage
Age	< 18	8	0.7
	18–25	348	32.3
	26–35	467	43.4
	36–45	209	19.4
	45–55	26	2.4
	> 55	19	1.8
Education	Primary	15	1.4
	Vocational	22	2.0
	Secondary	184	17.1
	Student	238	22.1
	Higher	618	57.4
Place of residence	Country	203	18.8
	Small village (< 50 k residents)	150	13.9
	Town (50 k–100 k residents)	120	11.1
	City (100 k–500 k residents)	103	9.6
	City (> 500 k residents)	501	46.5
Average income per household member	< 1000 PLN	89	8.3
	1000–2000 PLN	276	25.6
	2000–3000 PLN	317	29.4
	3000–4000 PLN	177	16.4
	> 4000 PLN	218	20.2
Marital status	Single	426	39.6
	Married	609	56.5
	Widow	9	0.8
	Divorced	33	3.1
Religion	Believer	845	78.5
	Non believer	232	21.5
Having children	Yes	715	66.4
	No	362	33.6
Being pregnant	Yes	110	10.2
	No	967	89.8
Having been pregnant	Yes	724	67.2
	No	353	32.8
Number of past pregnancies	0	351	32.6
	1	252	23.4
	2	294	27.3
	3	126	11.7
	4	37	3.4
	5+	17	1.6
Number of labours	0	368	34.2
	1	336	31.2
	2	284	26.4
	3	68	6.3
	4	14	1.3
	5+	6	0.6
Having had multiple pregnancy	Yes	80	7.4
	No	997	92.6
Having blood disease running in the family	Yes	102	9.5
	No	975	90.5

## Awareness

The overall awareness of cord blood banking was rather high in the studied population. Out of the 1077 women who participated in the survey, 84.6% (n = 911) admitted they had heard about umbilical cord blood banking. Women who had not heard about UCB were more likely to be under the age of 25 or over the age of 45, single, educated only up to high school or undergraduate (Table 2). Those who were aware of umbilical cord blood banking mostly had one or more children (n = 679, 74.5%). Among the women who were pregnant during the research (n = 110, 10.2%), the vast majority had heard about UCB banking before (n = 106, 96.4%). The place of residence and the average income per household member had no significant impact on women’s awareness of UCB banking (Table 2).

**Table 2:** Participants’ awareness of the umbilical cord blood banking depending on the age, education, marital status, place of residence and average income per household member

Category	Variables	Aware n (%)	Not aware n (%)	*p* value
Age	< 25	231 (64.9%)	125 (35.1%)	< 0.00001
	26–35	446 (95.5%)	21 (4.5%)	
	36–45	199 (95.2%)	10 (4.8%)	
	> 45	35 (77.8%)	10 (22.2%)	
Education	Primary	8 (53.3%)	7 (46.7%)	< 0.00001
	Vocational	17 (77.3%)	5 (22.7%)	
	Secondary	149 (81.0%)	35 (19.0%)	
	Student	160 (67.2%)	78 (32.8%)	
	Higher	577 (93.4%)	41 (6.6%)	
Marital status	Single	298 (70.0%)	128 (30.0%)	< 0.00001
	Married, Widow or Divorced	613 (94.2%)	38 (5.8%)	
Place of residence	Country	172 (84.7%)	31 (15.3%)	0.813
	Small village (< 50 k residents)	122 (81.3%)	28 (18.7%)	
	Town (50 k- 100 k residents)	102 (85.0%)	18 (15.0%)	
	City(100 k- 500 k residents)	89 (86.4%)	14 (13.6%)	
	City (> 500 k residents)	426 (85.0%)	75 (15.0%)	
Average income per household member	< 1000 PLN	73 (82.0%)	16 (18.0%)	0.779
	1000–2000 PLN	233 (84.4%)	43 (15.6%)	
	2000–3000 PLN	267 (84.2%)	50 (15.8%)	
	3000–4000 PLN	155 (87.6%)	22 (12.4%)	
	> 4000 PLN	183 (83.9%)	35 (16.1%)	

Out of the women who were aware of umbilical cord blood banking, only 124 (13.6%) had received the information from their physicians, while most of them (n = 433, 47.5%) had received it through the social media (Table 3). The subjective assessment of the general knowledge of umbilical cord blood banking was poor, as the majority (n = 563, 61.8%) assessed their level of it to be insufficient.

**Table 3:** Sources of information on umbilical cord blood banking

Source	How women obtained the information n (%)	How women would like to obtain the information n (%)
Physician	124 (13.6%)	791 (86.8%)
Nurse/midwife	119 (13.1%)	424 (46.5%)
Antenatal classes	250 (27.4%)	290 (31.8%)
Family/friends	189 (20.7%)	46 (5.0%)
Social media	433 (47.5%)	151 (16.6%)
TV/radio	152 (16.7%)	132 (14.5%)
Leaflets/posters/banners	254 (27.9%)	172 (18.9%)

In response to the question about how they would like to obtain the information about umbilical cord blood banking, women mostly indicated the doctor (n = 791, 86.8%) (Table 3).

Among the respondents who had known about cord blood banking, less than half (n = 383, 42%) was aware of the existence of both private and public UCB banks in Poland, 50.3% (n = 458) had heard only about private banks and 7.7% (n = 70) only about public ones. The majority was aware of the current research and therapeutic options in which umbilical cord blood is used. Most of them (n = 606, 89.8%) indicated haematological disorders such as leukaemias and lymphomas, 47.1% (n = 318)—stem cells defects, 39.3% (n = 265)—cancers, 28.9% (n = 195)—hereditary diseases, 24.9% (n = 168)—multiple sclerosis, 20.7% (n = 140)—cerebral palsy and 12.0% (n = 81) chose inherited metabolic disorders. 25.9% (n = 236) of the women were uncertain about the possibilities of umbilical cord blood usage.

## Attitude

Amongst the women who admitted that they had heard of UCB banking, 70% (n = 638) supported it. Potential possibility of helping their own child (n = 599, 93.9%) and helping other relatives (n = 411, 64.4%) were the most frequent reasons. Helping people who are not related (n = 253, 39.7%) and donating UCB for the scientific purposes (n = 127, 19.9%) were less popular. Respondents, who were not supporting UCB storing (n = 73, 8%), gave the high cost associated with private UCB banking as a main disadvantage.

Women, who had already stored or indicated willingness to store the UCB (n = 269, 29.5%), considered the following reasons as the most vital: potential possibility of helping their child (n = 258, 95.9%), helping other relatives (n = 186, 69.1%) or helping people that they were not related to (n = 87, 32.3%). Even though, only 33 (3.6%) participants had donated or intended to donate their child’s umbilical cord blood for the scientific purposes. The vast majority (n = 719, 78.9%) when asked, could also do it, if UCB unit did not meet the biomedical criteria required for other purposes. Around three-quarters of women (n = 670, 73.5%) agreed that UCB should be donated to a public bank every time it is not going to be stored in a private one.

**Fig. 1:**
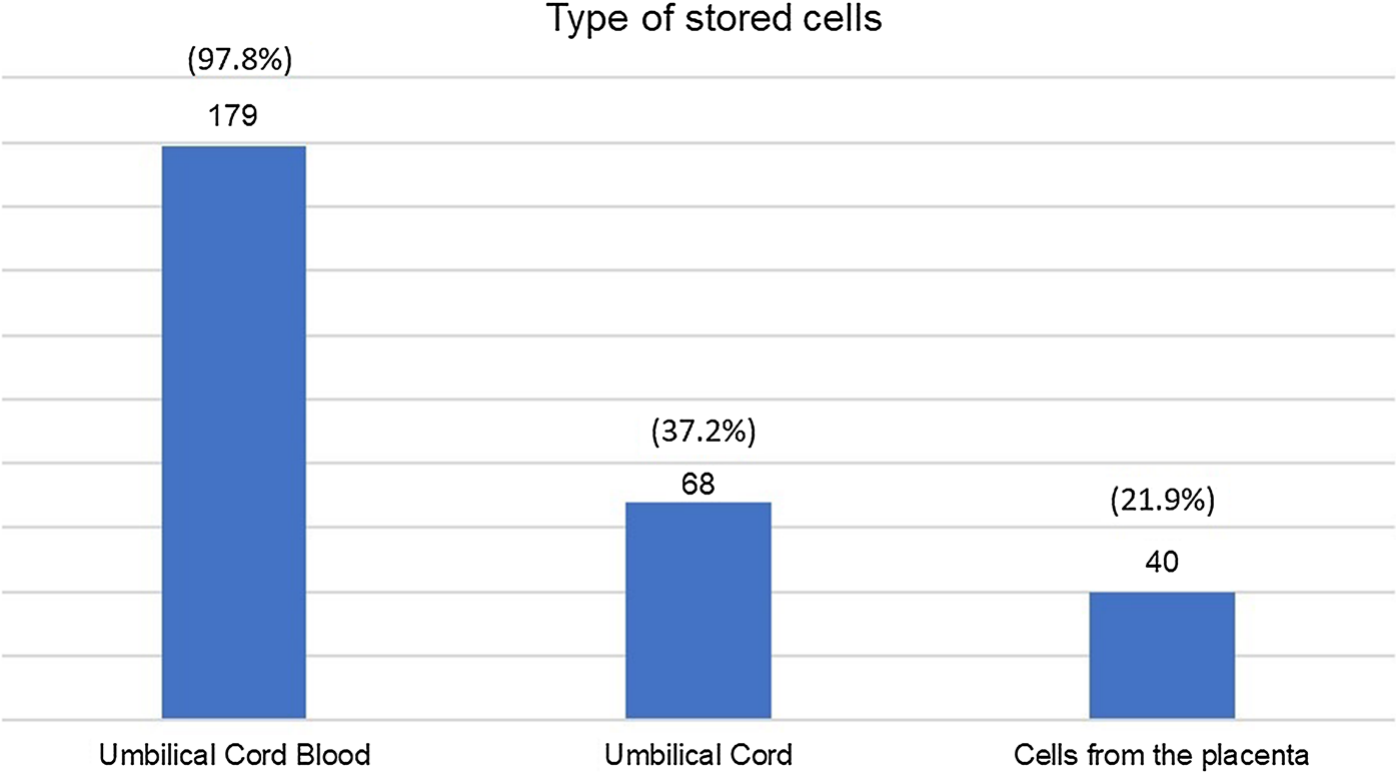
Type of stored cells (n = 183)

Among respondents who were willing to store or had already stored UCB, 47.6% (n = 128) preferred storage in a private bank, 9.3% (n = 25) in a public one and the rest was unsure or considered storage in both of them in case of more than one pregnancy. Having been asked about the type of cells that the participants are willing to store or have already stored, 183 (68.0%) of them answered; most of them pointed cord blood cells (n = 179, 97.8%) (Fig. 1).

More than half (n = 316, 66.9%) of the respondents, who had not stored and were not willing to store their children’s UCB (n = 472, 51.8%), indicated the high cost of UCB banking as the main reason of not collecting it. Amongst all of the women that were aware of UCB banking, 58% (n = 528) agreed that if the costs of private banking had been lower, they would have had their baby’s umbilical cord blood stored that way.

Table 4 presents the association between socio-economic and obstetric characteristics of respondents and their attitude towards storage of UCB. As it is shown in Table 4, both single and nulliparous participants admitted higher support regarding UCB storage.

**Table 4:** Association between socio-economic and obstetric characteristics of respondents and their attitude towards storage of UCB

Category	Variables	Positive attitude n (%)	Negative attitude n (%)	No opinion n (%)	*p* value
Age	< 18	3 (100.0%)	0 (0.0%)	0 (0.0%)	Not significant
	18–25	172 (75.4%)	13 (5.7%)	43 (18.9%)	
	26–35	290 (65.0%)	43 (9.6%)	113 (25.3%)	
	36–45	147 (73.9%)	16 (8.0%)	36 (18.1%)	
	46–55	19 (82.6%)	0 (0.0%)	4 (17.4%)	
	> 55	7 (58.3%)	1 (8.3%)	4 (33.3%)	
Education	Primary	5 (62.5%)	1 (12.5%)	2 (25.0%)	Not significant
	Vocational	13 (76.5%)	0 (0.0%)	4 (23.5%)	
	Secondary	103 (69.1%)	12 (8.1%)	34 (22.8%)	
	Student	126 (78.8%)	8 (5.0%)	26 (16.3%)	
	Higher	391 (67.8%)	52 (9.0%)	134 (23.2%)	
Place of residence	Country	128 (74.4%)	9 (5.2%)	35 (20.4%)	Not significant
	Small village (< 50 k residents)	90 (73.8%)	9 (7.4%)	23 (18.9%)	
	Town (50 k–100 k residents)	62 (60.8%)	11 (10.8%)	29 (28.4%)	
	City (100 k–500 k residents)	64 (71.9%)	8 (9.0%)	17 (19.1%)	
	City (> 500 k residents)	294 (69.0%)	36 (8.5%)	96 (22.54%)	
Average income per household member	< 1000 PLN	50 (68.5%)	8 (11.0%)	15 (20.6%)	Not significant
	1000–2000 PLN	162 (69.5%)	18 (7.7%)	53 (22.8%)	
	2000–3000 PLN	187 (70.0%)	18 (6.7%)	62 (23.2%)	
	3000–4000 PLN	108 (69.7%)	11 (7.1%)	36 (23.2%)	
	> 4000 PLN	131 (71.6%)	18 (9.8%)	34 (18.6%)	
Marital status	Single	235 (78.9%)	19 (6.4%)	44 (14.8%)	0.0002
	Married	385 (66.4%)	50 (8.6%)	145 (25.0%)	
	Widow	1 (14.3%)	1 (14.3%)	5 (71.4%)	
	Divorced	17 (65.4%)	3 (11.5%)	6 (23.1%)	
Religion	Believer	513 (71.1%)	49 (6.8%)	160 (22.2%)	0.028
	Non believer	125 (66.1%)	24 (12.7%)	40 (21.2%)	
Having children	Yes	465 (68.5%)	60 (8.8%)	154 (22.7%)	Not significant
	No	173 (74.6%)	13 (5.6%)	46 (19.8%)	
Being pregnant	Yes	62 (58.5%)	10 (9.4%)	34 (32.1%)	0.017
	No	576 (71.6%)	63 (7.8%)	166 (20.6%)	
Having been pregnant	Yes	470 (68.0%)	63 (9.1%)	158 (22.9%)	0.029
	No	168 (76.4%)	10 (4.6%)	42 (19.1%)	

No significant relationship between age, education, place of residence, income, and support for public UCB banking was found.

## Expectations from UCB banking

**Fig. 2:**
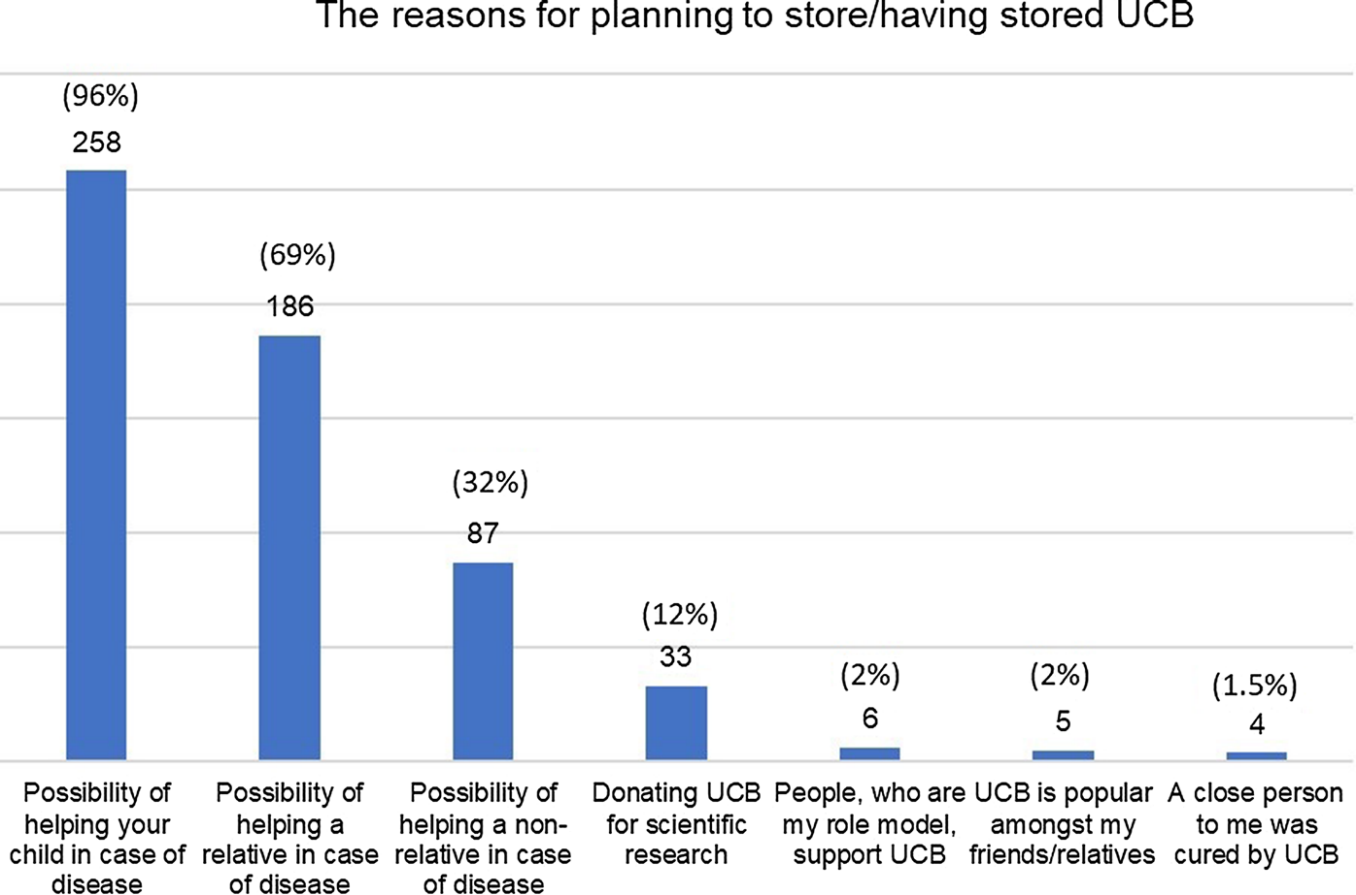
The reasons for planning to store/ having stored UCB (n = 269)

**Fig. 3:**
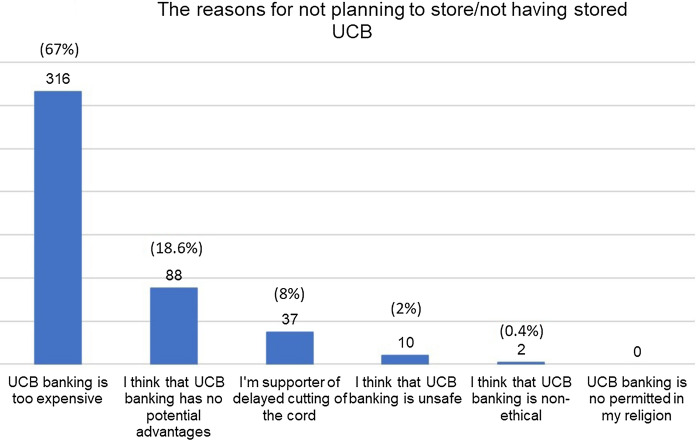
The reasons for not planning to store/ not having stored UCB (n = 472)

Among the participants who completed the fourth section of the survey, 557 women answered the question regarding the usefulness of UCB for its donor. 71.1% (n = 396) found this statement true, 12% (n = 67) disagreed with it and 16.9% (n = 94) admitted to have insufficient knowledge. However, only 269 (29.5%) of 911 participants banked UCB or were planning to do so. The reason of UCB banking are mainly the possibility of helping their children (n = 258, 95.9%) or relatives (n = 186, 69.1%) in case of any disease (Fig. 2). 316 of 472 women (66.9%) did not consider UCB banking due to the high costs of its storage (Fig. 3).

## Discussion

The study was conducted to explore Polish women's awareness and attitudes towards cord blood banking. Having been distributed in 46 Polish Facebook groups, the questionnaire-based study gathered information about women’s knowledge and opinion from different areas of the country and various marital and economic status. According to the data collected in late 2019, there were 18 350 000 Facebook users in Poland, including 9 725 500 women (NapoleonCat.com 2019). This represents almost 50% of the female population in Poland (Ambroch et al. 2019). The Internet is an enormous source of information on many topics and using the Internet can also influence the level of knowledge on UCB banking. We are aware that this is a survey conducted entirely online and this may affect the results. However, such form of research allowed us to obtain opinions from a much wider and more diverse group of women.

In the present research we established that even though the overall awareness regarding UCB banking was high among the studied group of women (n = 911, 84.6%), the subjective assessment of the general knowledge on umbilical cord blood banking was unsatisfactory (n = 563, 61.8%). This inadequate knowledge is consistent with other studies, such as a previous study conducted in 5 European countries. The authors showed that more than three-quarters of pregnant women declare having a poor knowledge of UCB (Katz et al. 2011). Similar results were seen in a study conducted in Greece, where only 48% of the participants had a full knowledge about UCB donation and storage (Karagiorgou et al. 2014), in a study performed in Beirut more than half of the women reported no prior knowledge (Saleh 2019).

The attitude towards UCB donation was mostly positive and most women were more likely to store UCB in a private bank. The main reasons of choosing private ones included potential possibility of helping their child and helping other relatives or people that they are not related to. On the other hand, women, who were against UCB banking, mentioned high cost as a common cause. 58% (n = 528) of all women agreed that if the costs of private banking had been lower, they would have had their baby’s umbilical cord blood stored that way.

UCB is known to be useful in haematological malignancy in children. However, the likelihood of using personal cord blood for the treatment of haematopoietic disorders before the age of 20 years ranges from 1/20,000 to 1/2700 (RCOG 2006). In our study 74.1% (n = 675) of respondents, who were aware of UCB banking, found UCB stem cells useful; they mostly indicated haematopoietic disorders (n = 606, 89.8%), stem cells defects (n = 318, 47.1%) and cancers (n = 265, 39.3%).

The majority of respondents, as in a study held in Hong Kong, expressed the opinion that they would prefer to be informed about umbilical cord blood banking by the physician, and the results showed that less than 15% received this information in that way (Suen et al. 2011). Having regard to a study conducted by Bhandari et al., which suggests that even a small amount of basic information can make a difference to women’s decisions about UCB banking, we should encourage medical personnel in Poland to pass the knowledge to their patients (2017).

The survey showed that the Internet is the main source of information for the majority of participants (n = 433, 47.5%). We suggest increasing the number of reliable websites where interested women could gain the latest knowledge about UCB banking.

What is worth knowing is the fact that most women (n = 670, 73.5%) agreed that UCB should be donated to public banks every time it is not going to be stored in private ones. Maybe this idea should be considered by the authorities in order to create a vast system of public UCB banks for the society’s needs.

## Conclusion

The knowledge and awareness regarding the possibilities of umbilical cord blood storage and banking among women in Poland is still low. Study shows that medical personnel, especially doctors, should provide reliable information to pregnant women. However, the general attitude towards UCB banking is highly-promising.
